# Wearable Devices for Arrhythmia Detection: Advancements and Clinical Implications

**DOI:** 10.3390/s25092848

**Published:** 2025-04-30

**Authors:** Ahmed Abdelrazik, Mahmoud Eldesouky, Ibrahim Antoun, Edward Y. M. Lau, Abdulmalik Koya, Zakariyya Vali, Safiyyah A. Suleman, James Donaldson, G. André Ng

**Affiliations:** 1 Department of Cardiovascular Sciences, Clinical Science Wing, Glenfield Hospital, University of Leicester, Leicester LE3 9QP, UK; ahmed.abdelrazik@leicester.ac.uk (A.A.); mie7@leicester.ac.uk (M.E.); ia277@leicester.ac.uk (I.A.); el203@leicester.ac.uk (E.Y.M.L.); aik8@leicester.ac.uk (A.K.); z.vali@leicester.ac.uk (Z.V.); sas97@leicester.ac.uk (S.A.S.); jad72@leicester.ac.uk (J.D.); 2 Department of Cardiology, University Hospitals of Leicester NHS Trust, Leicester LE3 9QP, UK; 3 NIHR Leicester Cardiovascular Biomedical Research Centre, Leicester LE3 9QP, UK; 4 Leicester British Heart Foundation Centre of Research Excellence, Leicester LE3 9QP, UK

**Keywords:** arrhythmia, arrhythmia detection, wearable devices

## Abstract

Cardiac arrhythmias are a growing global health concern, and the need for accessible, continuous monitoring has driven rapid advancements in wearable technologies. This review explores the evolution, capabilities, and clinical impact of modern wearables for arrhythmia detection, including smartwatches, smart rings, ECG patches, and smart textiles. In light of the recent surge in commercially available wearables across all categories, this review offers a detailed comparative analysis of leading devices, evaluating cost, regulatory approval, model specifications, and system compatibility. Smartwatches and patches, in particular, show a strong performance in atrial fibrillation detection, with patches outperforming Holter monitors in long-term monitoring and diagnostic yield. This review highlights a paradigm shift toward patient-initiated diagnostics but also discusses challenges such as false positives, regulatory gaps, and healthcare integration. Overall, wearable devices hold significant promise for reshaping arrhythmia management through early detection and remote monitoring.

## 1. Introduction

### 1.1. Overview of Arrhythmias and Need for Continuous Monitoring

Cardiac arrhythmias represent various heart rhythm irregularities, from sluggish to rapid, erratic to abnormal beats. They stem from diverse mechanisms and underlying triggers. While some arrhythmias may remain silent or inconsequential, others pose grave health threats, potentially culminating in severe complications such as stroke, heart failure, and even sudden cardiac death. Among the spectrum are notable conditions like atrial fibrillation (AF), ventricular tachycardia (VT), ventricular fibrillation (VF), supraventricular tachycardia (SVT), sinus bradycardia/pauses, and atrioventricular (AV) block. The symptoms linked with these abnormal rhythms can profoundly disrupt patients’ daily lives and well-being [1,2]. This was especially evident in studies from the developing world [3,4,5,6,7,8,9].

Additionally, the economic toll on healthcare systems due to arrhythmias, particularly AF, is staggering, estimated to reach as high as USD 26 billion annually in the United States alone [10]. Early and accurate detection is therefore essential for timely intervention and improved outcomes. However, traditional diagnostic tools such as 12-lead electrocardiograms (ECGs) and Holter monitors are often limited by short monitoring durations and user discomfort, failing to capture transient or asymptomatic episodes.

In response, the market has seen a rapid expansion in wearable technologies, ranging from smartwatches and smart rings to ECG patches and smart textiles, designed to provide continuous, real-time rhythm monitoring. Despite this technological boom, clinicians and healthcare providers face challenges in understanding the clinical utility, reliability, and limitations of the variety of available wearables. This review addresses this critical gap by providing a comprehensive, comparative evaluation of contemporary wearable devices used in arrhythmia detection. It offers detailed insights into their technological mechanisms, diagnostic performance, regulatory approval status, system compatibility, cost, and clinical applicability—helping guide evidence-based adoption in practice and policy.

### 1.2. History and Development of ECG

**Figure 1:**
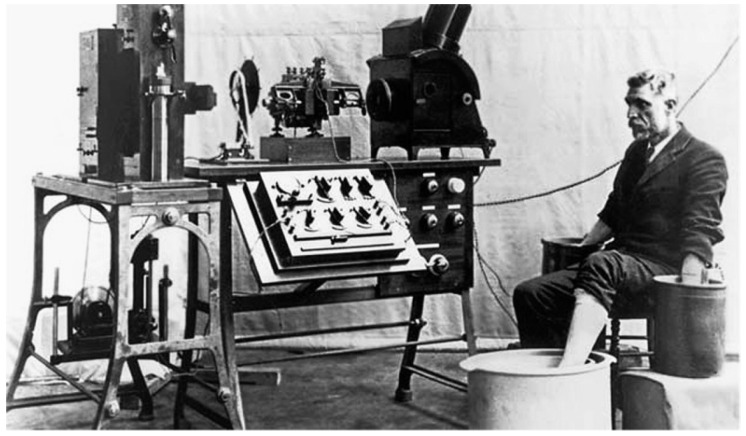
String galvanometer machine recording ECG.

Before the invention of the ECG, diagnosing arrhythmias was based on physical examination and auscultation, which lacked precision and often failed to detect subtle or intermittent abnormalities. The introduction of the ECG revolutionised cardiology by providing a detailed and reproducible representation of the heart’s electrical activity [11]. ECG has long been regarded as the gold standard for diagnosing arrhythmias, a status maintained for over a century. The history of the ECG began in the early 20th century, when Dutch physiologist Willem Einthoven invented the first practical electrocardiograph in 1903. Einthoven’s innovative work, which earned him the Nobel Prize in 1924, relied on using a string galvanometer to record the heart’s electrical impulses (Figure 1). This breakthrough allowed for the visualisation of heart rhythms, enabling clinicians to detect abnormal patterns indicative of various cardiac conditions [12].

Early models of the ECG were large and cumbersome, but they quickly improved in portability and accuracy. The ECG evolved from an analogue tool to a highly sophisticated digital instrument as technology progressed. The development of the standard 12-lead ECG system further refined diagnostic capabilities, allowing clinicians to capture a comprehensive view of the heart’s electrical function from different angles. Hence, ECG can detect the slightest changes in electrical activity and direction, crowning it as the gold standard equipment in detecting various kinds of arrhythmia with incomparable precision [13].

Recording arrhythmias has long been challenging, but recent years have seen significant progress. Achieving a rhythm/symptom correlation is crucial for effectively treating patients with these conditions. While ECGs are the cornerstone for diagnosing arrhythmias, the main hurdle has always been recording the arrhythmia in action. Some arrhythmias occur sporadically, while others go unnoticed by the patient. This has led to the need for continuous monitoring to catch these elusive events [14,15].

### 1.3. Development of Continuous Monitoring Device: Holter Monitor

While it is the gold standard test in diagnosing arrhythmia, 12-lead ECG provides a snapshot of the heart’s electrical activity at a moment, limiting its usefulness in arrhythmia detection, which naturally presents in paroxysms. The Holter monitor, introduced by Dr. Norman Holter in the early 1940s, emerged as a solution to this limitation, offering continuous monitoring of the heart’s electrical activity over an extended period, ranging from 24 to 48 h. This allowed for the detection of transient arrhythmias that might be missed during a 12-lead ECG recording. Early Holter devices struggled with signal quality, battery life, and data storage issues. Moreover, noise interference and poor electrode adhesion to the skin also impacted the reliability of long-term monitoring [16,17].

**Figure 2:**
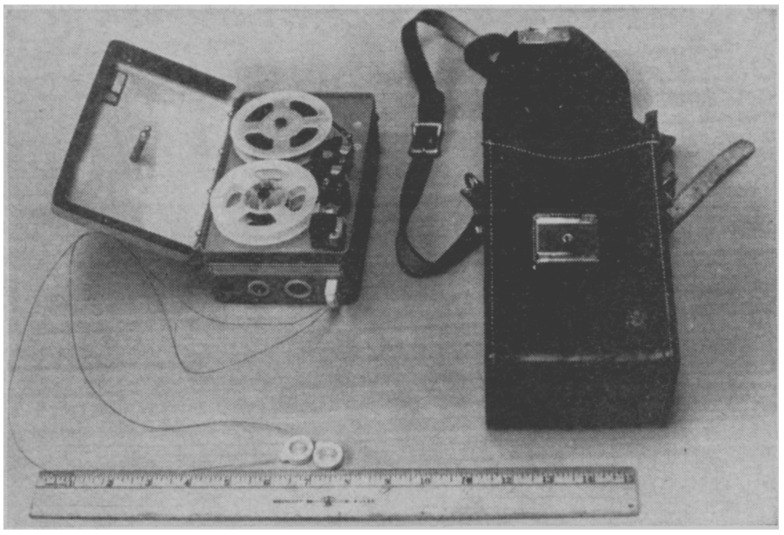
Old Holter monitor unit showing tape recorder, electrodes, and carrying case.

By the 1970s, innovations in electronics allowed for a reduction in the number of leads used in Holter monitors (Figure 2). Transitioning to three-lead systems (often called single-channel or three-channel ECG) enabled more comfortable and wearable devices. This reduction in lead number simplified the design, made it easier for patients to tolerate the device, and reduced the system’s weight. The 3-lead Holter monitor provided the advantage of continuous monitoring of the heart’s basic rhythm, though it could capture fewer details compared to the 12-lead system. Despite these limitations, the three-lead system was highly effective in detecting common arrhythmias, particularly AF, ventricular ectopic beats, and tachycardias, which were the primary focus of these devices [18].

By the late 1980s and 1990s, modern Holter monitors were much smaller, lightweight, and capable of transmitting data wirelessly for remote analysis. Digital technology allowed for more precise signal processing and accuracy in arrhythmia detection [18]. Despite significant advancements in Holter monitor technology, these devices continue to present challenges for patients in their daily lives. The electrode patches used for attachment to the skin can irritate, particularly during extended wear. Patients often encounter difficulties maintaining normal daily activities, such as dressing or showering, while wearing the device, due to the limitations imposed by the electrodes and the associated hardware. The wires connecting the electrodes to the recording unit further restrict the patient’s range of motion, which may interfere with their daily routines.

Moreover, the practical use of Holter monitors is typically limited to a maximum duration of 7 days, which can still miss less frequently presenting arrhythmias in some patient populations who can have their symptoms once a month or a few times per year [19,20].

### 1.4. Advancements in Wearable Devices in Enhancing Arrhythmia Detection and Management

**Figure 3:**
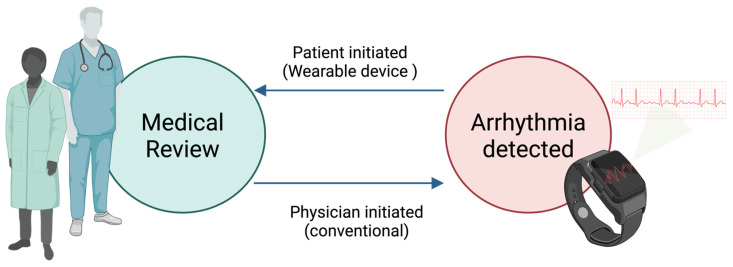
Wearable devices inverted the paradigm of healthcare delivery.

In recent years, there have been significant technological advancements in wearable devices, particularly smartwatches, for detecting arrhythmias. Despite being marketed as consumer gadgets, these advanced wearables can measure health data comparable to ambulatory electrocardiographic monitors. One notable advantage of these devices is their ability to provide continuous monitoring with minimal restrictions; some are equipped with advanced technologies capable of automatically detecting irregular, slow, or fast abnormal rhythms. This capability is particularly crucial for identifying infrequent and asymptomatic arrhythmias, thereby aiding in managing the affected individuals. However, this advantage comes with the risk of oversensing certain arrhythmias, potentially leading to unnecessary anxiety for the wearer and unnecessary hospital visits. Wearable devices have achieved an unprecedented milestone in the healthcare system’s history; they have inverted the paradigm of healthcare delivery (Figure 3). Contrary to the usual, where the patient seeks medical advice and presents to a health care practitioner for further investigations of their symptoms, with wearable devices detecting arrhythmias, those patients seek medical services after their devices detect possible arrhythmias and inform them of them [21].

### 1.5. Objectives of This Review

To provide practitioners with a summary of the most recent developments in wearable technologies with cardiological applications, this review offers the present state of wearable technologies, including smartwatches and patch monitors. It delves into their technological diversity, clinical utilities, and prospective trajectories, providing a nuanced understanding of their roles in contemporary healthcare.

## 2. Smartwatches

### 2.1. Introduction to Smartwatch

In recent years, watches have transformed from mere timekeeping accessories to sophisticated devices that facilitate communication, allow easy smartphone access, and gather health data comparable to medical-grade equipment. These include heart rate (HR), ECG, heart rate variability (HRV), and blood oxygen saturation. The smartwatch market is growing exponentially. In 2021, the industry was valued at USD 59 billion and is expected to be worth USD 100 billion by 2025 [22].

Smartwatches should be assessed alongside other rhythm-monitoring devices to determine if they qualify as medical devices, considering clinical factors such as the length of monitoring, continuous tracking, and the ability to record events or symptoms on demand. Nonclinical considerations like ease of use, invasiveness, and cost should also be considered. Current guidelines and expert consensus documents acknowledge the utility of smartwatches in certain clinical settings, such as screening for AF in high-risk groups and measuring the AF burden to aid in managing the condition. Furthermore, recent and ongoing research is exploring the use of smartwatches for detecting and managing other arrhythmias, which could broaden their clinical applications [23,24]. Incorporating ECG functionality in smartwatches presents a remarkable opportunity for continuously monitoring patients with sporadic arrhythmias, exhibiting commendable diagnostic accuracy. This capability holds promise for reducing the strain on healthcare services attributable to recurrent hospital visits and inconclusive diagnostic procedures that fail to detect arrhythmic events.

### 2.2. Smartwatch Technology

Most smartwatches utilise photoplethysmography (PPG) technology to generate heartbeat signals and detect irregularities underlying arrhythmias. PPG is a non-invasive technology that smartwatches use to monitor the heart rate by detecting changes in blood volume through the skin. The device typically uses a light source directed onto the skin. The light penetrates the skin and reflects off the blood vessels. A photodetector then measures how much light is reflected, which varies with the pulsatile flow of blood with each heartbeat [25].

The PPG signal comprises two components: One component detects blood volume. When the heart beats, the blood volume in the vessels increases, which causes a corresponding change in the amount of light absorbed or reflected. By continuously measuring these variations, the smartwatch can determine the heart rate, and it can also assess the regularity or irregularity of the heartbeats; Hence, giving notification to the user for the possibility of irregular heart rhythm and prompting the user to take an ECG, if provided by the wearable device [26].

The other component is influenced by respiration, temperature, and autonomic nervous system activity. Consequently, PPG can furnish additional detailed data post-processing through machine learning algorithms, encompassing parameters such as ECG, HRV, and oxygen saturation. PPG is valued for its simplicity, non-invasiveness, low cost, versatility, and accuracy, rendering it widely employed in smartwatches. Nonetheless, its limitations are notable and recurrent, including susceptibility to sensor displacement from the skin surface; significant movement artefacts, particularly pronounced during physical exertion; and susceptibility to ambient noise interference [25].

### 2.3. Comparative Analysis of PPG and ECG Technologies in Wearable Devices

When assessing wearable devices for arrhythmia detection, understanding the fundamental differences between PPG and ECG technologies is crucial. ECG directly measures the heart’s electrical activity through electrodes placed on the skin, while PPG indirectly assesses cardiovascular function by detecting blood volume changes in the microvasculature. From a technical standpoint, ECG remains the gold standard for arrhythmia diagnosis due to its ability to capture detailed electrical waveforms (P waves, QRS complexes, and T waves), enabling the precise identification of specific arrhythmias and conduction abnormalities. Clinical-grade ECG typically uses multiple leads to view cardiac electrical activity from different angles comprehensively. In contrast, wearable ECG implementations, particularly in smartwatches, are limited to fewer leads (often single-lead), which restricts their diagnostic capabilities compared to 12-lead clinical ECG systems. PPG technology offers simplicity, non-invasive advantages, and requires only a single point of contact, making it ideal for continuous monitoring in consumer devices. However, PPG has inherent limitations for arrhythmia detection. A systematic review by Pereira et al. (2020) found that PPG-based AF detection achieved a pooled sensitivity of 91.6% (95% CI: 87.9–94.3%) and specificity of 95.9% (95% CI: 93.1–97.7%), which is lower than ECG-based detection [27]. The primary technical challenges of PPG include the following: (1.) Indirect measurement: PPG detects pulses rather than electrical activity, making subtle arrhythmias harder to detect. (2.) Motion artefacts: Physical activity significantly affects the signal quality, with considerable signal degradation during exercise. (3.) Physiological variables: Skin perfusion, ambient temperature, and autonomic tone can all affect PPG’s signal quality. (4.) Temporal resolution: PPG has a lower temporal resolution than ECG, limiting the detection of brief arrhythmic events. Recent technological innovations have attempted to address these limitations through multi-sensor fusion approaches. Devices incorporating PPG and ECG capabilities, such as the Apple Watch and Samsung Galaxy Watch, implement a two-stage detection system: continuous PPG monitoring for irregular rhythm alerts and on-demand ECG recording for confirmation. This hybrid approach leverages the continuous monitoring advantages of PPG with the diagnostic precision of ECG when needed. Tison et al. (2018) demonstrated that passive monitoring using PPG technology in commercially available smartwatches could identify AF with modest accuracy, showing the potential of such approaches for population screening [28]. When selecting a wearable device for arrhythmia monitoring, clinicians should consider that PPG-only devices may be suitable for screening and flagging potential issues. In contrast, ECG-capable wearables provide more definitive diagnostic information that closely aligns with clinical-grade cardiac monitoring.

**Figure 4:**
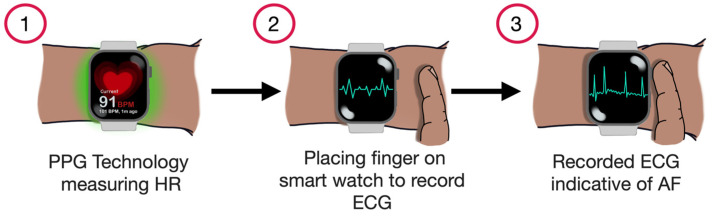
Demonstrating the process of AF detection using a smartwatch.

The latest generation of smartwatches can also generate a single-lead ECG by utilising the watch’s body and crown as two distinct electrodes. When worn on the left wrist and with one of the right-hand fingers placed on the crown, the device generates a waveform corresponding to lead I of a conventional 12-lead ECG (Figure 4). However, it has limitations, especially for unstable arrhythmias. For instance, capturing data requires a 30 s recording window, which can be inconvenient. Moreover, environmental noise and movement of the arm during recording may lead to errors or false readings [29,30]. Another possibility is that, by varying the smartwatch’s position on different body parts, it is possible to capture waveforms replicating other leads of the standard 12-lead ECG [31].

### 2.4. Different Smartwatches and AF Detection

#### 2.4.1. Apple Watch

The Apple Heart Study was a large-scale, siteless study conducted with 419,297 participants over eight months, assessing the potential of a smartphone app and smartwatch-based irregular pulse notification system to detect AF [32]. Participants without prior self-reported AF used an Apple iPhone app to consent to passive monitoring through a smartwatch. If an irregular pulse was detected, a telemedicine consultation was triggered, and participants were mailed an ECG patch to wear for up to seven days. Over a median monitoring period of 117 days, only 0.52% (2161 participants) received irregular pulse notifications. Among the 450 participants who returned an ECG patch with analysable data, AF was detected in 34%, with a similar rate (35%) in those aged 65 or older. Importantly, the irregular pulse notifications’ positive predictive value (PPV) was high, 0.84 for detecting AF through ECG at the time of notification and 0.71 for tachogram concordance with ECG findings. These findings suggest that the smartwatch notification system is fairly reliable in identifying true AF cases, with 84% of notifications accurately reflecting AF [32].

#### 2.4.2. Fitbit

The Fitbit Heart Study enrolled 455,699 participants between May and October 2020 to assess the effectiveness of the Fitbit wearable devices’ algorithm in AF [33]. Irregular heart rhythms occurred in 1% of participants, and 4% of those aged 65 and older, over a median follow-up period of 122 days. Among the 1057 participants who received an irregular heart rhythm notification and returned an ECG patch with analysable data, 32.2% were found to have AF. The study showed a high PPV of 98.2% and 97% for participants ≥65 years old [33].

#### 2.4.3. Samsung Galaxy

In a study conducted with 200 participants, out of 100 patients with sinus rhythm (SR), 81 ECGs were correctly diagnosed as SR, 6 were misclassified as AF, and 13 were unclassified [34]. For 100 patients with AF, 88 were correctly diagnosed, 5 were wrongly classified as SR, and 7 were inconclusive. When unclassified ECGs were considered false, the sensitivity was 88% (95% confidence interval [CI]: 80–94%), the specificity 81% (95% CI: 72–88%), with a PPV of 82% and a negative predictive value (NPV) of 87%. Excluding unclassified ECGs, the sensitivity rose to 94% (95% CI: 87–98%) and specificity to 94% (95% CI: 87–98%), with a PPV of 95% and an NPV of 93% [34]

#### 2.4.4. Withings ScanWatch Horizon

In a study conducted on 262 participants, after the exclusion of dropouts, blinded, board-certified cardiologists independently reviewed the 12-lead reference ECGs and the single-lead ECGs recorded by the smartwatch [35]. Three separate reviewers evaluated each ECG recording. To assess the performance of AF detection, the reviewers’ classifications were compared to the algorithm’s automatic classification of the single-channel ECG strips recorded by the smartwatch. The software categorised these strips into four groups: normal sinus rhythm, AF, noise, or other arrhythmias. The algorithm’s performance in differentiating AF from normal sinus rhythm was assessed in two ways: first, by including all four initial categories (sensitivity 0.770; specificity 0.965), and second, by excluding the “Other” and “Noise” categories (sensitivity 0.963; specificity 1.000). Misclassification rates were very low for patients with normal sinus rhythm or AF. Notably, the false positive rate for normal sinus rhythm was 2.7% (3 out of 113), and no cases of AF were mistakenly identified as normal sinus rhythm by the algorithm [35].

#### 2.4.5. Huawei Smartwatches

The mAFA-II Trial screening for AF was conducted across China using PPG technology in smart devices, including the Honor Band 4 and Huawei Watch GT, for individuals aged 18 and older [36]. Participants wore the devices for at least 14 days. Healthcare providers further validated episodes of “possible AF” detected by the PPG algorithm through clinical evaluation, ECG, or 24 h Holter monitoring at the MAFA Telecare centre and network hospitals. A total of 187,912 individuals used PPG smart devices to monitor their pulse rhythm between 26 October 2018, and 20 May 2019. Out of the 187,912 individuals monitored, 424 (0.23%, mean age 54 years, 87.0% male) received a “suspected AF” notification. Among those who were followed up, 227 out of 262 individuals (87.0%) were confirmed to have AF, resulting in a positive predictive value of 91.6% for PPG signals (95% CI: 91.5–91.8%). Both suspected and confirmed cases of AF significantly increased with age (p for trend <0.001), with the highest rate of detected AF found in Northeast China at 0.28% (95% CI: 0.20–0.39%) [36].

#### 2.4.6. Garmin Smartwatches

A study enrolled 200 consecutive participants, who underwent ambulatory Holter ECG monitoring to detect atrial AF or evaluate AF burden. Simultaneously, participants were monitored for 24 h using a Garmin smartwatch with continuous PPG recording [37]. PPG signals were processed for noise elimination, beat detection, beat classification, and rhythm analysis in 5 min intervals. The accuracy of AF detection from the PPG recordings was assessed by comparing it with the simultaneous Holter ECG, which served as the diagnostic reference for AF. Of the 200 participants, 112 (56%) experienced AF, forming the AF group. The detection of AF in these participants showed a sensitivity of 97.3%, a specificity of 88.6%, and a PPV of 91.6%. When AF detection was evaluated in those processed 5 min segments, the sensitivity was 97.1%, the specificity was 86.8%, and the PPV was 89.7% [37].

#### 2.4.7. Other Arrhythmia Detection

Multiple studies have examined the effectiveness of smartwatches in detecting arrhythmias and ECG abnormalities beyond AF, which is the most frequently studied arrhythmia in this context. This emphasis on AF is due to its intermittent nature and the significant health risks it poses, particularly the increased risk of stroke [38]. However, as smartwatch technology advances, these devices have a growing potential to detect other arrhythmias accurately. In these cases, the smartwatch can identify abnormalities such as significantly elevated or reduced heart rates, prompting the user to initiate a manual ECG recording via the device, if available. These ECG recordings can then be reviewed by a healthcare professional, potentially providing valuable insights into arrhythmias, particularly those that are intermittent or transient. This detection method allows for capturing sporadic arrhythmic events that might otherwise go undetected, significantly enhancing early diagnosis and management and improving patient outcomes.

In a study on 260 patients who underwent standard 12-lead ECG, the Apple Watch Series 4 generated four single-lead ECGs. These were recorded by placing the watch on different body parts. The left wrist corresponded to Lead I, the left ankle corresponded to Lead II, the right parasternal area represented Lead V1, and the left midaxillary line in the fifth intercostal space represented Lead V6. Two blinded cardiologists reviewed these recordings from both groups, and any conflict was to be resolved by a third blinded cardiologist.

In bradyarrhythmias, the Lead I recording from Apple Watch was very efficient in diagnosing sinus bradycardia, with a sensitivity of 93% and a specificity of 98%. However, the same lead could only detect 7/10 cases of complete AV block and 0/3 of second-degree AV block. When the other recorded leads (Leads II, V1, V6) were added, it enabled the detection of all cases of complete AV block and two out of three of the second-degree AV block.

On the other hand, as expected, the Apple Watch could accurately detect AF with a 96% sensitivity and 91% specificity. However, it was low for detecting atrial flutter (AFL) or atrial tachycardia (AT), with a 25% sensitivity and 99% specificity. Adding the other three recorded leads by the Apple Watch significantly increased the accuracy of diagnosing these arrhythmias, which reached a 69% sensitivity and 100% specificity [39].

Another Apple Watch Series 4 and 5 study was conducted on 256 patients compared to a standard 12-lead ECG. Two blinded cardiologists reviewed the recordings from the 12-lead ECG and the Apple Watch ECG. Sinus bradycardia and AV block were correctly diagnosed in 81% of participants, and two patients with VT were correctly identified on the Apple Watch ECG. As expected, the diagnostic accuracy for AF was high, with a sensitivity of 96% and a specificity of 91%. Still, these were low for AFL/AT, with a sensitivity of 25% and a specificity of 99%. However, AF and AFL/AT were correctly differentiated in 71% of cases [40].

Hwang and colleagues explored the accuracy of smartwatches in measuring HR in known patients with SVT. They asked participants to wear two smartwatches while conducting an electrophysiology study (EPS) for SVT, HR at baseline and during the arrhythmia was recorded. The Apple Watch Series 2, Samsung Galaxy Gear S3, and Fitbit Charge 2 were tested. The accuracy of baseline HR measurements (within ±5 beats per minute [bpm] of an ECG) was 100% for both the Apple and Galaxy devices and 94% for the Fitbit. During induced SVT, with HRs ranging from 108 bpm to 228 bpm, the accuracy (within ±10 bpm of an ECG) was 100% for the Apple, 90% for the Galaxy, and 87% for the Fitbit [41].

Multiple systematic reviews and meta-analyses have emerged in the past few years, proving the excellent diagnostic capabilities of recent smartwatches in detecting various types of arrhythmias, especially AF. The Apple Watch is FDA-approved and is the smartwatch most studied for detecting arrhythmias, with the greatest accuracy in detecting AF. A recent systematic review by Bogár et al., which included different types of smartwatches, showed that the Apple Watch™, the most frequently used, has an over 90% accuracy in AF detection and an around 90% sensitivity and specificity. Similar results were concluded with different types of smartwatches in detecting AF, including the Samsung Simband^®^, Samsung Galaxy Watch 3^®^, Huawei Watch GT 2 Pro^®^, and Fitbit Sense 2^®^. Besides detecting AF, these smartwatches showed reliability in detecting different bradyarrhythmias [42]. In another systematic review by Pay et al., exploring the smartwatch’s ability to detect arrhythmias other than AF, smartwatches provided reliable results in detecting brady/tachyarrhythmia, with a considerable ability in differentiating the type of arrhythmia. Specifically, AF was the single arrhythmia detected with the highest accuracy; there was low accuracy in distinguishing between atrial flutter and different atrial arrhythmias [26]. The key features of leading smartwatches are summarised in Table 1.

## 3. Smart Rings

### 3.1. Background

Since the 1990s, smart rings have been recognised as an ideal wearable device for digital technology due to their small size and convenience for long-term wear. Early prototypes were larger and impractical, but smart rings became more feasible for everyday use as design advancements took place. Over the years, with improvements in sensor technology and miniaturisation, smart rings have evolved into efficient health and fitness-monitoring [43] tools. Their small form factor and the ease with which they can be worn for extended periods make them a convenient alternative to larger wearables like smartwatches. Smart rings have gained popularity for continuous biometric monitoring due to their stability, ease of wear, and effective data collection capabilities. They offer advantages over wrist-worn devices, especially regarding their stable positioning and consistent skin contact on the fingers. Fingers have a dense network of blood vessels and robust blood flow, which is a crucial advantage when using PPG sensors for measuring vital signs [44]. These features make rings ideal for measuring the heart rate (HR), heart rate variability (HRV), oxygen saturation, respiratory rate, sleep data, and women’s health metrics (like cycle tracking and pregnancy insights [45]). A comparison of popular smart ring models is provided in Table 2.

### 3.2. Advantages of Smart Rings

#### 3.2.1. Stable Positioning and Skin Contact

Unlike wrist-worn devices like smartwatches, which are subject to motion artefacts due to wrist movements, smart rings offer a stable finger positioning, ensuring consistent and reliable data collection. This makes them ideal for continuous biometric monitoring.

#### 3.2.2. Enhanced Signal Clarity

The fingers have a dense network of blood vessels and strong blood circulation, which makes smart rings particularly effective when using PPG sensors. This anatomical advantage allows clearer signals when measuring heart rate, oxygen saturation, and other vital parameters [44].

#### 3.2.3. Continuous Health Monitoring

Smart rings can monitor various health parameters continuously without being intrusive. They can measure heart rate, HRV, sleep data, and oxygen levels. Some even track women’s health, providing insights into menstrual cycles and pregnancy [45].

#### 3.2.4. Compact and Comfortable

Their small size allows for a comfortable and unobtrusive fit, making them more convenient for users who want to track their health discreetly, compared to the bulkier smartwatches or fitness bands.

#### 3.2.5. Blood Pressure Estimation

PPG sensors in smart rings, like those used in some smart ring models, have been showing an emerging role in estimating blood pressure effectively in studies like those conducted by Wang et al. [46].

### 3.3. Disadvantages of Smart Rings

#### 3.3.1. Limited Functionalities Compared to Smartwatches

While smart rings excel in biometric data collection, they generally do not offer the broad functionality of smartwatches, such as GPS, notification display, or interactive features. They are primarily designed for health monitoring.

#### 3.3.2. No Built-In ECG for Some Models

Most smart rings do not have built-in ECG sensors, which limits their ability to detect more complex heart conditions such as arrhythmias or AF. This is a significant drawback for users looking to monitor AF directly from their wearable devices. A study by Gala et al. demonstrated that the CART-I ring, which can generate ECG, showed a higher sensitivity and specificity (86.6% and 89.4%, respectively) compared to the Apple Watch (67.4% and 74.9%, respectively) [47].

**Table 2:** Comparison of smart ring models focusing on cost, subscription fees, system compatibility, sensor features, and sizing.

Smart Ring	FDA	CE	Cost	Subscription Fee	PPG	ECG	System Compatibility	Sizes	Average Battery Life
Oura Ring 4 [45]	✕	✓	GBP 349	GBP 5.99/month	✓	✕	iOS and Android systems	4–15, no half sizes	7–8 days
Samsung Galaxy Ring [48]	✕	✓	GBP 399	None	✓	✕	Android system only	5–15, no half sizes	Up to 7 days
Ultrahuman Ring Air [49]	✕	✓	GBP 329	None	✓	✕	iOS and Android systems	5–14, no half-sizes	4–6 days
Amazfit Helio Ring [50]	✕	✓	GBP269	None	✓	✕	iOS and Android systems	8, 10, and 12 sizes only	1–3 days

**FDA**: U.S Food and Drug Administration, **CE**: Conformité Européene.

#### 3.3.3. Battery Life and Charging

The need for small, lightweight batteries in smart rings often leads to a limited battery life. While they can last several days, they typically require frequent charging compared to other wearables like fitness trackers or smartwatches.

#### 3.3.4. Comfort and Fit

Smart rings must be properly sized to ensure accurate readings. If the ring is too loose or too tight, the sensors may not work as effectively, which could potentially inconvenience users.

## 4. Patch Monitors

### 4.1. Overview

Adhesive ambulatory ECG patches are another innovative engineering product that challenges conventional short to medium-term ECG monitoring methods, particularly multi-lead Holter monitors. They typically comprise a sensor system, a microelectronic circuit with a recorder, memory storage, and an embedded internal battery. They are usually affixed to the body through a prefabricated adhesive material. Typically, they are lighter, wireless, waterproof, and minimally intrusive to daily activities. Their designs and characteristics allow them to achieve a longer average study period and completion rate per use episode than conventional Holter monitors [1]. Most patches have trigger buttons for symptomatic patients, allowing for symptom/rhythm correlation [51]. Their wireless capabilities have the potential to enable remote physician monitoring and immediate action in the event of a serious cardiac arrhythmia [52].

These devices can detect most clinically relevant arrhythmias, including sinus tachycardia, bradycardia, AF, atrial flutter, junctional rhythms, atrial ectopy, ventricular ectopy, ventricular tachycardia, pauses, and second- and third-degree heart blocks [1,2]. The traces obtained, however, do not necessarily correspond to conventional 12-lead ECG configurations [52]. Data from the patches is frequently analysed automatically using deep learning algorithms [53]. In many situations, these automated algorithms have demonstrated an excellent accuracy in interpreting the produced single-lead ECGS compared to 12-lead ECGs [54]. However, Koshy et al. found that around 15% of traces remained unclassified when studying the outcome of a single-lead ECG algorithm [55]. Therefore, a hybrid approach is needed, to use automated diagnoses offered by these devices in conjunction with additional clinical analysis [55]. Given the higher number of traces produced by these devices [52], this could create a greater workload for healthcare professionals.

Lately, these patches have become available directly to consumers even before going through the necessary clinical validation needed to assess their effectiveness, reliability, and safety by regulators. This is important, given that the data from these devices can extend beyond wellness tracking and fitness assessment to influence therapeutic and interventional decision-making. This direct-to-consumer approach remains attractive to the industry to reach a broad customer base. However, the resulting heterogeneity in collected data that rely on various devices and technologies has complicated their wide implementation into healthcare systems [51,52].

Some of these patches have sensors that can monitor other physiological parameters such as temperature, respiratory rate, and arterial oxygen saturation. This has the potential to significantly facilitate and improve remote patient monitoring, allowing timely investigations and treatment. This can enhance community-based care, which is currently being more widely adopted, given the nature of the growing elderly population with a higher chronic disease burden and the reality of hospital bed scarcity [52,56].

In a survey completed by over 400 physicians from 42 countries, most participants were positive about the ability of these devices to facilitate arrhythmia diagnostics and screening. However, concerns regarding data overload were raised; therefore, scientific societies’ recommendations were deemed necessary [57]. These technologies will involve exchanging sensitive patient health data through wireless networks; thus, regulators must also address the associated security and privacy concerns [51]. In the UK, the National Institute for Health and Care Excellence (NICE) has recommended using the ECG patch Zio^®^ XT for people with suspected cardiac arrhythmias who would benefit from ambulatory ECG for longer than 24 h. They advised that National Health Service (NHS) organisations using the device will be required to collect information on resource use and long-term clinical consequences. They have also emphasised that NHS organisations using Zio^®^ XT should ensure that the service complies with general data protection regulations (GDPRs) and that informed consent covers how a person’s data will be used [58]. Some of the available ECG patches are summarised in the Table 3. The information in this table only reflects the devices’ current characteristics and capabilities, as the technology is rapidly evolving. It might become outdated as soon as they are amended or upgraded by their manufacturers.

### 4.2. Reliability

ECG patches were used in several clinical trials to assess their reliability, feasibility, and cost, and they were even used as an already-established method for evaluating different management outcomes. Some of the following studies were carried out on the abovementioned devices and other devices. It is worth noting, however, that these devices are continuously being modified and upgraded, so their characteristics and capabilities at the time of these studies might not be the same as their current characteristics and capabilities.

As part of the SAFETY study, the Firstbeat™ Bodyguard 2 patch’s sensitivity, specificity, and overall accuracy in AF diagnosis achieved 96.3% (89.7% to 99.2%), 98.5% (96.6% to 99.5%) and 98.1% (96.3% to 99.2%), respectively, when compared with 12-lead ECG. The assessment was conducted in a single screening visit [78]. A systematic review by Hermans et al. analysed eight studies on different ECG patches. They found that the overall sensitivity and specificity of ECG patches in AF detection ranged between 93.4% and 97%, and 95.6% and 98.8%, respectively [31]. Campero Juardo et al. analysed the signal quality of the Vital Signs patch over 5 days and found no statistically significant signal degradation over time [79]. Jortveit et al. invited 40 general practitioners to review ten long-term ECGs generated by the ECG247 patch. Arrhythmias were correctly identified in most cases, with a sensitivity of 98%, specificity of 75%, and diagnostic accuracy of 89%. General practitioners rarely correct incorrect automatic system algorithm interpretations [80]. In summary, ECG patches achieved a high overall accuracy, with no significant signal degradation over time and good physician interpretability.

Several studies used ECG patches as a screening tool in asymptomatic patients. In the mSToPS randomised controlled trial, the actively monitored group with a home-based Zio^®^ XT patch had an AF detection rate of 6.7%, compared to 2.6% in the control group receiving routine care over 12 months. Compared to 4 months later, starting the monitoring immediately had improved AF detection by 3%. More patients were started on anticoagulation due to the increased detection rate [81]. The SCREEN-AF study recruited 856 participants who were not known to have AF. It randomised them to receive two episodes of clinic review and pulse check vs. two episodes of 14-day monitoring by ZIO^®^ XT patch. There was a 10-fold increase in AF detection in the group that received the patch, for 75% of whom anticoagulation was initiated [82]. Kwun et al. screened 320 non-AF patients using the AT Patch for 11 days, resulting in a new diagnosis of AF in 3.4% of them [83]. The SAFS study detected new AF in 1.7% of the 355 non-AF recruited patients using the Spyder patch for 3 to 14 days (median duration of 5.4 days) [84]. Using the Cardea SOLO™ patch, Lin et al. found that serial screening for AF in high-risk post-menopausal women with 7-day patches over 12 months increased AF detection from 2.5% at baseline to 3.7 at 6 months and 4.9% at 12 months. The yield was even higher among women with high CHARGE-AF scores at 4.2%, 5.9%, and 7.2%, respectively [85]. The SEARCH-AF study compared AF detection post-cardiac surgeries between a group monitored with ECG patches for 30 days vs. standard follow-up. The SEEQ patch was initially used in the first part of the trial, followed by the CardioSTAT patch in the latter part. The patients were equally randomised between the two groups, and out of the 33 patients detected to have AF, 29 were in the continuous patch-monitoring group [86]. Lee et al. utilised the HiCardi^®^ patch’s remote monitoring function to conduct a clinical trial on 2000 hikers. A total of 318 participants had abnormal ECG signals, out of whom 182 were advised to visit the hospital. The follow-up revealed that out of the 30 people who were confirmed to have visited the hospital as advised, 7 (23.33%) were diagnosed with cardiovascular disease [87]. In summary, when used as a screening tool, ECG patches improved arrhythmia detection rates in multiple clinical settings and patient populations.

ECG patches were also trusted to be used in clinical trials to assess end outcomes. Using the ZIO^®^ XT patch as a screening tool over two 14-day periods, Heo et al. concluded that CKD significantly increases the risk of AF incidence among individuals with diabetes [88]. The S-Patch EX was used by Chow et al. to monitor selected patients for 30 days, enabling them to identify the degree of arrhythmia burden on a group of COVID-19 survivors [89]. The Cardea SOLO patch enabled Gomez et al. to find a positive correlation between the frequency of PVCs and CHARGE-AF score in part of the WHISH trial population [90]. Data from the Zio^®^ patch has helped Wineinger et al. classify paroxysmal AF into subtypes that might differ in pathophysiology and stroke risk [91]. In summary, ECG patches have proved to be highly trusted in the clinical research field.

### 4.3. Performance

Several studies were also conducted to compare the performance of ECG patches with currently established modalities. Torfs et al. performed a cross-correlation analysis in a clinical validation study between the Imec patch and a 24 h Holter. The rhythms were obtained simultaneously on ten different patients with known AF. There was an 85% overall correlation between the raw data and up to 99% between R-R intervals. The performance of the Imec patch matched that of the 24 h Holter [92]. In another study by Hyun et al., 39 post-cardiac surgery patients were simultaneously monitored with a mobiCARE™ patch and Holter monitor for 24 h. The diagnostic accuracy was comparable, as both techniques identified paroxysmal AF in seven patients [93]. Ahn et al. compared the mobiCARE™ patch with a 24 h Holter regarding a PVC burden analysis. They simultaneously attached both to 134 patients. Both monitors agreed regarding the PVC burden, with a mean difference of −0.07% [94]. The SMART-TEL study compared the SmartCardia™ patch with the Holter monitor on 53 cardiac surgery patients for 48 h. The Holter identified 190 events, while the patch identified 174 events, of which cardiologists assessed 169 as true events. The number of false alarms in the Holter group was 21, compared to 5 in the patch group [95]. In summary, the ECG patch performance largely matched traditional Holter monitoring, with fewer false alarms.

The importance of extended cardiac ambulatory rhythm monitoring was highlighted by Schultz et al. when they retrospectively studied the arrhythmia incidence in 314 adult congenital heart disease patients. Using a Zio^®^ patch for a median duration of 9.5 days, significant arrhythmias were identified in 156 patients, of which only 46% were noted within 48 h [96]. In a systematic review, Yenikomshian et al. studied 23 publications with patients monitored by the Zio^®^ patch. The mean monitoring period in the included publications was 10.4 days, and the percentage of patients with detected arrhythmias within 48 h was less than 65%. In a large retrospective analysis of commercial data available from the Zio^®^ patch, Krumerman et al. concluded that the mean error in PVC detection reduced from 2.9% at 24 h to 1.3% at 7 days and 0.7% at 10 days, recommending extending the PVC burden analysis beyond 24 h to 7 days to improve its accuracy [97]. Extended ambulatory monitoring beyond the traditional 24 and 48 h results in better arrhythmia detection and a more accurate PVC burden assessment.

The EPACS study compared the AF detection rate between 14-day Zio^®^ patch monitoring vs. 24 h Holter monitoring in patients who recently had TIA or ischaemic stroke. The patch group had a paroxysmal AF detection rate of 16.3%, compared to 2.1% in the Holter group [98]. Ju Young Kim et al. compared arrhythmia detection rates on 58 participants simultaneously fitted with a 7-day MEMO^®^ patch and a 24 h Holter. The overall arrhythmia detection rate was 34.5% with the patch vs. 19% with the Holter [99]. In another study, Soohyun Kim et al. simultaneously fitted 59 paroxysmal AF patients with an 11-day AT-Patch and a 24 h Holter to assess their response to anti-arrhythmic drugs. AF or atrial tachycardia were detected in 47.5% of the patch group vs. 13.6% of the Holter group [100]. Chen et al. compared 14-day EZYPRO^®^ patch monitoring vs. a 24 h Holter by simultaneously attaching both in 83 patients who had either ischaemic stroke or syncope. The arrhythmia detection rate of the patch was 69.9% vs. 21.7% for the Holter [101]. In summary, 7- to 14-day patch ECG monitoring achieves higher arrhythmia detection rates than 24 h Holter monitoring.

### 4.4. Feasibility

The feasibility of using extended ambulatory ECG monitoring by Zio^®^ patch in outpatient screening for asymptomatic AF was tested in the STUDY-AF trial. The outcome was positive, with a mean wear time of 10.4 days and an AF detection rate of 5.3% [102]. Khan et al. proved the same in their feasibility study in a stroke clinic setting. The mean wear time of the Zio^®^ XL patch was 12.1 days, with a 3.9% paroxysmal AF detection rate [103]. Pothineni et al. tested the feasibility of ECG patch monitoring by posting the Zio^®^ XT patch to selected participants of the REGARDS study. The mean wear time was 12.9 days, and 2 of the 15 patients were found to have AF [104]. In Lang et al.’s feasibility study, the median Zio^®^ XT patch wear time was 13.9 days, and 3.5% of patients were found to have AF. The use of the patch has resulted in fewer hospital visits and an overall positive patient experience, with 82% of patients welcoming the use of the patch again [105]. A study by Tomonori et al. using an eMemo patch has demonstrated good tolerability in an elderly population after a heart procedure, in which the aortic valve was replaced through a percutaneous approach, with a mean wear time of 12.4 days. A total of 152 arrhythmia events were detected in 198 patients, warranting therapeutic interventions, including pacemaker implantation for an AV block and anticoagulation for newly diagnosed AF [106]. Patrick et al. tested the feasibility of ECG patch monitoring in 95 patients with developmental and intellectual disabilities. The mean wear time was 12.2 days, and 30.5% of participants received a new cardiac diagnosis [107]. The feasibility of fully digital self-screening for AF was tested by Sandberg et al. using the ECG247 patch, achieving a mean monitoring time of 153 h and a number needed to screen of 45 [108]. In summary, ambulatory monitoring by ECG patches was feasible and tested in multiple clinical settings and patient populations.

### 4.5. Cost

Regarding the cost implications, the EPACS study’s economic model showed that the higher AF detection rate of 14 h monitoring by the Zio^®^ patch compared to the 24 h Holter can result in yearly savings of up to GBP 113,630, rising to GBP 162,491 in 5 years. This results in an estimated 10.8 strokes avoided per year [98]. A cost analysis of the mSToPS study outcome by Reynolds et al. has concluded that despite a healthcare cost increase resulting from the active monitoring strategy, AF screening using 2-week patches was associated with a high economic value. The quality-adjusted survival difference resulted in an incremental cost-effectiveness ratio of USD 36,100 per quality-adjusted life year gained, which is considered high value by US cardiac professional societies [109]. In their cost analysis study, Medic et al. found that the initial strategy of 30-day MCOT patch monitoring before implantable loop recorder (ILR) insertion achieved eight times lower costs than proceeding directly to ILR, when the insertion purpose was for AF detection post-stroke. An estimation of 50–60% will not need to proceed with the ILR insertion following the 30-day MCOT monitoring period [110]. In summary, ambulatory monitoring by ECG patches can be of a high economic value.

### 4.6. Additional Uses

Ambulatory ECG patches have demonstrated they can have additional uses in healthcare. In their proof-of-principle study, Verrier et al. utilised the Preventice BodyGuardian MINI-EL patch to track the post-MI risk of sudden cardiac death. The study proposes that ECG patches play a future role in assessing that risk and guiding management through monitoring T-wave alternans (TWA) and T-wave alternans heterogeneity (TWH) measurements [111]. The novel Jewel P-WCD (patch–wearable cardioverter–defibrillator) is an example of the potential therapeutic uses of ECG patches. In a study by Hummel et al., 305 patients who were not eligible or declined ICD were enrolled. The patch delivered 11 shocks in total, and 9 of them were appropriate. The median daily wear time was 23.5 h [112]. In summary, the rapidly evolving field of ECG patches can extend beyond ambulatory monitoring.

### 4.7. Challenges

The GUARD-AF study tried to assess the potential healthcare effect of 14-day AF screening by the Zio^®^ XT patch. AF was diagnosed in 5% of the screening group vs. 3.3% in the usual care group, and anticoagulation was started in 4.2% vs. 2.8%, respectively. There was no impact on hospitalisation. However, these results were statistically underpowered, as the recruitment terminated prematurely after enrolling only 11,905 patients of the initial target of 52,000 due to the impact of the COVID-19 pandemic. Additionally, anticoagulation was only started in 55% of the newly diagnosed AF patients in the study. Further studies are needed to evaluate the net clinical benefit of AF screening [113].

Routinely implementing ECG patches in healthcare comes with its challenges. One of the main mentioned advantages of ECG patches is reducing the number of hospital visits, given their self-application potential. In a study by Goergen et al., the return rate of the Zio^®^ XT patch in the self-applying group was 90.8% vs. 97.1–98.1% in the clinic-applied group [114]. When Sandberg et al. conducted their AF digital screening feasibility study in a care home for the elderly, they faced multiple recruitment challenges; 26% of the assessed individuals were not included in the study due to severe cognitive impairment and 36% due to lack of willingness to participate. However, using the ECG247 patch for AF screening in the participating group remained feasible [115]. The analysis of signals from long-term wearable devices also has the potential to be labour-intensive, given that the absolute amount of signal noise will increase by routinely increasing the duration of monitoring. Continuously improving noise detection algorithms is essential to reduce clinical technicians’ time and effort in correctly classifying these noises [116].

## 5. Smart Shirts

Smart shirts, also known as smart textiles, e-textiles, e-shirts, and smart vests, represent a significant advancement in wearable technology, designed to monitor various health parameters. The integration of sensors into clothing began in the early 2000s, driven by a growing health-conscious population. Early developments focused on embedding sensors into garments to monitor basic activity parameters such as heart rate and motion, aimed at providing continuous monitoring for various populations, including healthy individuals, high-risk patients, post-event patients, and athletic professionals [117].

By the mid-2010s, there was a significant evolution in smart shirt technology, transitioning toward the incorporation of ECG sensors. This transition marked a pivotal change in arrhythmia detection. With ECG monitoring, smart shirts began enabling the real-time continuous tracking of the heart’s electrical activity, offering a non-invasive and accessible method for detecting conditions such as AF [118].

In recent years, these smart shirts have further evolved, particularly in professional and athletic applications. They are now widely used to monitor athletes’ health, track activity levels, and even measure advanced parameters such as respiratory functions. Additionally, they have proven valuable in medical contexts, serving as reliable ECG monitors for arrhythmia detection [119]. Table 4 presents a comparison of selected smart shirts.

### 5.1. Clinical Evidence for Smart Shirts in Arrhythmia Detection

Despite their technological promise, smart shirts require rigorous clinical validation to establish their role in arrhythmia detection. Recent clinical trials have begun to establish an evidence base for these wearable textiles in cardiac monitoring applications. Steinberg et al. (2019) evaluated a novel wearable ECG recording device embedded in a garment against conventional Holter monitoring [120]. Their proof-of-concept study demonstrated a comparable signal quality between the smart shirt and Holter recordings, with 95% of the ECG segments recorded by the wearable device being of sufficient quality for rhythm analysis. Importantly, patients reported greater comfort with the textile-based system compared to traditional Holter monitors, suggesting the potential for improved adherence during long-term monitoring [120]. Seshadri et al. (2019) conducted a comprehensive review of wearable sensors for monitoring athletes, including textile-based ECG systems [120]. Their analysis highlighted that smart shirts equipped with ECG sensors could effectively capture cardiac rhythms during physical activity with minimal motion artifacts compared to adhesive electrodes, providing a promising platform for arrhythmia detection in active individuals. However, they noted significant technical challenges, including the need for improved electrode designs to maintain skin contact during movement [121]. The integration of bio-impedance monitoring into smart textiles represents another advancement in arrhythmia detection capabilities. Blachowiz et al. (2021) demonstrated a comprehensive review of novel 3D sewn electronics approaches, which achieved a varying accuracy in detecting cardiac and respiratory anomalies compared to standard medical devices [122]. Some smart textile systems incorporate dedicated artifact reduction channels, allowing for an improved signal quality during activities of daily living [122]. Addressing the technical challenge of developing multi-lead ECG capabilities in textiles, Boehm et al. (2016) created an innovative 12-lead ECG T-shirt with active electrodes [123]. Their validation against the standard 12-lead ECG demonstrated a strong correlation (r = 0.94) between the systems for key cardiac measurements. The T-shirt design maintained a consistent electrode positioning, addressing a common limitation of standard ECG monitoring, where electrode placement variability can affect the diagnostic accuracy [123]. These empirical findings suggest that smart shirts represent a technically viable approach for arrhythmia monitoring, with a diagnostic performance approaching that of conventional systems. Their strength lies in enabling extended monitoring periods with greater patient comfort, potentially increasing the diagnostic yield for intermittent arrhythmias. However, current evidence highlights the need for further technical refinement to improve the signal quality during activity and address usability challenges, particularly in elderly populations.

### 5.2. Advantages of Smart Shirts

**Comfortable Fit and Sensor Placement**: One of the primary benefits of smart shirts is their good fit over the upper body, offering ample space for sensors. This allows for the placement of multiple sensors in optimal positions, closer to vital organs, which enhances the accuracy of signals and monitoring [124].

**Continuous ECG Monitoring**: Smart shirts enable continuous, real-time ECG monitoring, which is essential for detecting arrhythmias and tracking the heart rate over long periods. This long-term monitoring is critical for patients with heart conditions, allowing for the early detection of AF and other irregular heart rhythms [118].

**Versatility for Different Populations**: These garments serve a wide range of users, including athletes, healthcare providers, and high-risk patients. Their ability to offer continuous monitoring in a non-invasive manner makes them ideal for healthcare follow-ups and preventative care.

**Convenience and Comfort**: Unlike bulky ECG machines, smart shirts are wearable and designed for daily use, allowing for continuous monitoring without the inconvenience of medical devices. They are designed to be comfortable and non-restrictive, offering a user-friendly experience.

### 5.3. Disadvantages of Smart Shirts

**Limited Clothing Flexibility**: As smart shirts are specifically designed for health monitoring, they can be limiting in terms of clothing choices. Wearing a smart shirt regularly may prevent individuals from considering other clothing options, limiting their fashion versatility.

**Durability and Washing**: Smart shirts require careful attention to durability and maintenance. As these garments include electronic components, their washability and ability to withstand repeated washing cycles are crucial for long-term usability. If the garment is not durable enough, it can deteriorate quickly with regular wear and washing [118].

**Battery Life**: Smart shirts rely on battery-powered sensors to function, and the battery life of these garments can be a limiting factor. Frequent charging may be required, and ensuring that the battery lasts long enough to provide continuous monitoring throughout the day is a critical consideration. A recent study by De Fazio et al. proposed new solutions that would allow extended periods of battery life by recovering energy from the human body [125].

**Table 4:** Comparison of some smart shirts by cost, battery life, monitored parameters, and device compatibility.

Smart Shirt	FDA	CE	Cost	Parameters	Compatibility	Average Battery Life
**Hexoskin Smart Shirt** [126]	✕	✓	USD 249	Cardiac (ECG, HR, HRV, and QRS events), respiratory, activity, and sleep data	iOS and Android Systems	12–24 h
**Cardioskin** [127]	✓	✓	Not commercially available, intended for clinical use	12-lead continuous ECG, HR, activity, and respiratory data	N/A	N/A
**Athos Smart Shirt** [128]	✕	✕	USD 398	No ECG, targeted more towards activity and respiratory data. Measures muscle activity through EMG	iOS Systems only	6–12 h

**FDA**: U.S Food and Drug Administration, **CE**: Conformité Européene, **EMG**: Electromyography.

**Cost and Accessibility**: The price of smart shirts can be a barrier for some users. Advanced features such as ECG monitoring and respiratory functions may drive up the cost, making these shirts more expensive than traditional garments or even some medical devices.

**Algorithm Architectures in Wearable Arrhythmia Detection:** The evolution of algorithms for arrhythmia detection in wearable devices has progressed from simple rule-based approaches to sophisticated machine learning models. Understanding this algorithmic taxonomy is essential for clinicians to evaluate devices’ performance and reliability. Current approaches can be categorised into four main architectures, each with distinct characteristics and performance profiles.

1. **Traditional Rule-Based Algorithms** were the first generation of detection systems used in early wearable monitors. These algorithms employ predefined thresholds and decision trees based on established electrophysiological parameters, such as RR interval variability, to detect arrhythmias. While computationally efficient and interpretable, their performance is limited by rigid decision boundaries that may not accommodate the physiological variability across different patient populations.
2. **Feature-Based Machine Learning** approaches extract hand-crafted features from the cardiac signal (e.g., heart rate variability metrics, wavelet coefficients, spectral characteristics) and employ classifiers such as support vector machines or random forests. These algorithms offer an improved performance over rule-based systems, with Wasserlauf et al. (2019) reporting a sensitivity of 94.3% and specificity of 95.8% for AF detection in smartwatch data [29]. However, their effectiveness remains dependent on the quality of feature engineering and may not generalise well across different populations.
3. **Deep Learning Neural Networks** represent the current state of the art in wearable arrhythmia detection. Hannun et al. (2019) demonstrated a deep neural network that could classify 12 rhythm classes from a single-lead ECG with a performance exceeding that of cardiologists [129]. Their network achieved an F1 score exceeding 0.80 for detecting AF, atrial flutter, and other arrhythmias, demonstrating the potential of these approaches for the automated interpretation of data from wearable monitors.
4. **Hybrid and Ensemble Systems** combine multiple algorithmic approaches to improve robustness. These systems often employ a two-stage architecture: a computationally efficient algorithm for continuous screening and a more sophisticated algorithm for detailed analysis when irregularities are detected. Torres-Soto and Ashley (2020) demonstrated that multi-task deep learning approaches that simultaneously detect multiple cardiac abnormalities can improve performance by leveraging shared signal characteristics across different arrhythmias [130].

Recent innovations focus on addressing key challenges in real-world deployment. Attia et al. (2019) developed an AI-enabled ECG algorithm capable of identifying patients with AF during sinus rhythm, demonstrating the potential to detect underlying cardiac abnormalities even when arrhythmias are not actively present [131]. This approach could significantly enhance the value of periodic ECG recordings from wearable devices by identifying patients at risk of developing arrhythmias before they occur. The performance of these algorithms varies considerably across arrhythmia types. Current systems excel at AF detection, most achieving a >90% sensitivity and specificity in controlled studies. However, performance remains suboptimal for other arrhythmias, particularly those with complex or subtle ECG manifestations. These limitations highlight the need for a more comprehensive validation across the full spectrum of arrhythmias and in diverse patient populations.

## 6. Conclusions

Integrating wearable devices into arrhythmia detection presents an exciting frontier in cardiovascular healthcare. Their availability enables patient-initiated monitoring, allowing individuals to begin tracking their health independently before engaging with healthcare providers, facilitating earlier interventions and potentially improving outcomes. These devices extend beyond arrhythmia detection by offering data on VO_2_ max, oxygen saturation, respiratory rate, and body temperature, enabling a more holistic view of health. As the wearable technology market grows, these tools become more accessible; however, meaningful clinical integration still poses significant challenges.

One major concern is the limited validation of wearable devices for detecting arrhythmias beyond AF. Data reliability, especially during physical movement, remains inconsistent, raising questions about these devices’ accuracy and clinical utility in real-world conditions. Overdiagnosis due to false positives can result in unnecessary healthcare utilisation and patient anxiety, underscoring the need for improved specificity through more advanced, rigorously tested algorithms.

Furthermore, while regulatory approval by bodies such as the FDA and CE indicates baseline safety, direct-to-consumer ECG technologies’ broader ethical and legal implications are less clear. As patients increasingly interpret health data independently, clearer definitions of clinician responsibility and liability are needed, alongside strategies to enhance patients’ understanding of potentially complex results.

Data privacy adds another layer of complexity. With devices continuously collecting sensitive biometric information, strict adherence to regulations such as the GDPR is essential. Manufacturers and healthcare providers must ensure transparent, secure data handling practices to maintain user trust and regulatory compliance.

Cost remains a significant barrier. Many wearable devices, particularly smartwatches, are still financially out of reach for underserved populations, risking the deepening of existing health disparities. Strategies such as insurance reimbursement models, public–private partnerships, and community-level initiatives are vital to broaden equitable access.

Future research should prioritise refining detection algorithms through machine learning, which holds promise for improving the specificity of arrhythmia detection and reducing the burden of false positives. Sophisticated AI models trained on large, diverse datasets could help distinguish true pathological rhythms from benign anomalies or artefacts caused by movement. Additionally, integrating multi-modal biosignals—such as combining ECG data with photoplethysmography, accelerometry, and even voice or sweat analysis—could enhance the diagnostic accuracy and contextual understanding of the user’s physiological state.

Targeted strategies are required to mitigate digital inequity. These may include designing lower-cost, clinically validated alternatives to premium smartwatches, expanding access through insurance reimbursement or national health schemes, and providing digital literacy programmes to ensure users can effectively engage with the technology. Public health initiatives could also distribute wearables to high-risk populations as part of preventive cardiovascular screening programmes, potentially reducing the incidence of adverse cardiac events.

Furthermore, developing interoperable platforms that seamlessly integrate wearable data into electronic health records would enhance clinician access to continuous health information and support more informed decision-making. Implementing clear standards for data interpretation, accountability, and patient consent will be crucial to navigating the medico-legal complexities of incorporating wearable data into clinical care.

By addressing these gaps, wearable technology can evolve from a consumer novelty into a trusted, scalable tool in cardiovascular medicine. This would not only improve early detection and patient engagement but also contribute to a more proactive, data-driven, and equitable model of healthcare delivery.

## Figures and Tables

**Table 1:** The key features of leading smartwatches include regulatory status, cost, AF detection capability, core studies, and sensor technology.

Manufacturer	Model	FDA	CE	Cost *	Af Detection Automatically **	Core Study	PPG	ECG	Average Battery Life ***
Apple Inc.	Series 4, 5, 6, 7, 8, 9, 10 ULTRA 1 and 2	✓	✓	GBP 300–800	✓	Apple Heart Study	✓	✓	18–36 h
Alphabet Inc.	Fitbit Sense and Sense 2 Fitbit Charge 5 and 6	✓	✓	GBP 150–400	✓	Fitbit Heart Study	✓	✓	2–7 days
Samsung Electronics	Galaxy Watch Active 2. Galaxy Watch 3,4,5,6,7	✓	✓	GBP 250–400	✓	-	✓	✓	1–3 days
Withings	Withings Move ECG ScanWatch ScanWatch Horizon ScanWatch Light	✓	✓	GBP 250–400	✓	-	✓	✓	8–30 days
Huawei Technologies Co.,	GT 3 Pro Gt 5 Pro 4 Pro Space Edition	✕	✓	GBP 300–500	✓	mAFA-II Trial	✓	✓	5–9 days
Garmin Ltd.	Venu 2 plus Venu 3 Series Epix Pro Fenix 7 Pro	✓	✓	GBP 350–700	✓	-	✓	✓	3–10 days

* Prices vary depending on the model. ** Alerts user of signs of irregular heartbeats and offers to use the ECG feature to confirm *** Average battery use depends on model, features used, and personal use. **FDA**: U.S Food and Drug Administration **CE**: Conformité Européene.

**Table 3:** This overview of selected ECG patch monitors includes wear duration, features, ECG lead configuration, regulatory status, usage setting, and additional monitoring capabilities.

Device	Summary
ZIO^®^ XT by iRHYTHM [59]	- Single-use cardiac monitoring patch powered by AI technology - Could be worn for up to 14 days - Available through clinician prescription - The patient wears the monitor, then posts it back to iRhythm^®^ - Zio^®^ provides clinicians with a report of the findings. Preliminary findings are generated by a machine learning algorithm, then validated by certified cardiac physiologists - Recommended by NICE
HiCardi^®^ SmartPatch^®^ [60]	- Single-lead ECG monitoring - Can also monitor respiratory rate, temperature, and detect falls and sleep apnea - Reusable - Battery operation time (72–168 h), charging time (1–2 h) - Up to 7 days’ memory - Web-based software to allow remote monitoring of multiple patients - For use by qualified healthcare professionals
SmartCardia™ [61]	- 7-lead ECG patch - Can last for 14 days - Offers real-time ECG and vitals monitoring through a cloud platform - Reusable across patients and does not require charging - Clinician-controlled dispensing
Jewel^®^ Wearable Defibrillator [62]	- By Element Science - Not currently commercially available for sale - Provides a defibrillation function, as well as 24/7 monitoring
ECG247™ by Appsens AS [63]	- Commercially available for consumers, as well as healthcare professionals - Gives immediate feedback when heart rhythm disturbances are detected - Data uploaded directly to secure cloud storage - Monitors for up to 7 days - Can be used multiple times
EZYPRO^®^ by SIGKNOW [64]	- Lasts for up to 14 days - Available through physician prescription - Device is returned to EZYPRO^®^ by patient for analysis - Report is generated by EZYPRO^®^’s certified analysts and sent to physician
Firstbeat™ Bodyguard 2 [65]	- Attached to the skin with two electrodes - Battery lasts for 6 days and is rechargeable. Charge time is 1 h - Memory capacity is 20 days - For fitness professionals’ use
Imec’s Health Patch [66]	- Disposable patch - Can also measure respiratory rate and oxygen saturation - The collected information is processed on-chip and then transmitted into a mobile phone or the cloud through an integrated Bluetooth connection - Can provide 7–14 days of acquisition - Can provide real-time data visualisation through an Android app - Novel modality for building next-generation tools
Cardea SOLO™ [67]	- By Cardiac Insight - Provides monitoring for up to 7 days - Comprised of a sensor, a smart USB cable for PC connectivity, and software that automatically generates draft summary reports within 5 min - Available on prescription from healthcare provider
Philips MCOT ™ [68]	- The MCOT monitor gathers data from the sensor by Bluetooth and sends them automatically via a wireless connection - Available on prescription from physician - The monitor is sent back to the company for analysis. A report is generated and sent to the physician and patient - Patches last around 5 days. The sensor can be recharged, enabling up to 30 days of wear
mobiCARE™ by Seers [69]	- Can provide monitoring for up to 9 days - ECG analysed by a deep learning AI algorithm - Final report provided by an arrhythmia specialist from mobiCARE™ - Available on prescription from healthcare providers
MEMO^®^ Patch 2 by HUINNO [70]	- Can provide monitoring for up to 14 days - Data can be uploaded and managed through a dedicated app - Available on prescription from medical staff - The patch is handed back to the hospital, and ECG analysis reports will be available to healthcare staff through the dedicated MEMO^®^ health platform
Spyder Wireless ECG [71]	- Can provide an extended diagnostic ECG as well as remote patient monitoring - Can provide monitoring for up to 30 days - Allows a remote real-time review through a web-based dashboard - Reports can be generated by the core lab remotely and verified by certified cardiologists - Available on prescription by physician
AT Patch by ATsens [72]	- Can provide monitoring for up to 14 days - Real-time ECG monitoring of patients admitted to a ward is available - Disposable after single use - Report can be generated by dedicated software - Commercially available for consumers
VitalPatch^®^ by VitalConnect [73]	- Connects to a dedicated relay device - Enables near-real-time remote vital signs streaming through a cloud-based algorithm - Can also measure respiratory rate and temperature and detect falls - Provides streamlined ECG diagnostic reports - Single use. Lasts for up to 7 days - Available on prescription from physician
CardioSTAT^®^ [74]	- Can last up to 14 days - Available through prescription by healthcare providers - The patient posts the device back at the end of the monitoring period - A report is generated and certified by cardiac physiologists, then is made available to the referring physician
S-Patch ExL by Wellysis [75]	- Provides real-time ECG remote monitoring - Data are synced to a mobile or watch and are shared with the medical team via a cloud - Reports generated using cloud-based algorithm - Can last for up to 11 days - Reusable after battery change
BodyGuardian™ MINI [76]	- By Boston Scientific - Allows near-real-time remote cardiac monitoring - Available on prescription by doctor - Can be worn for up to 15 days - Fully rechargeable - Preliminary reports generated through deep learning AI algorithms. Can be edited by technicians
Philips ePatch^®^ [77]	- Can last up to 14 days - Reports generated through AI cloud-based software and validated by cardiologists - Battery is rechargeable and reusable - Available on prescription from physician
